# Evaluating the Appropriateness of Downscaled Climate Information for Projecting Risks of *Salmonella*

**DOI:** 10.3390/ijerph13030267

**Published:** 2016-02-29

**Authors:** Galina S. Guentchev, Richard B. Rood, Caspar M. Ammann, Joseph J. Barsugli, Kristie Ebi, Veronica Berrocal, Marie S. O’Neill, Carina J. Gronlund, Jonathan L. Vigh, Ben Koziol, Luca Cinquini

**Affiliations:** 1 National Climate Predictions and Projections platform (NCPP), NCAR RAL CSAP, 3450 Mitchell Lane, Boulder, CO 80301, USA; ammann@ucar.edu; 2 Department Atmospheric, Oceanic and Space Sciences, University of Michigan, 525 Space Research Building, Ann Arbor, MI 48109-2143, USA; rbrood@umich.edu; 3 CIRES—NOAA/University of Colorado, 325 Broadway, Boulder, CO 80305-3328, USA; joseph.j.barsugli@noaa.gov (J.J.B.); ben.koziol@noaa.gov (B.K.); 4 Department of Global Health, School of Public Health, University of Washington, 1959 NE Pacific Street, Health Sciences Building, Seattle, WA 98195, USA; krisebi@uw.edu; 5 Department of Biostatistics, University of Michigan School of Public Health, 1415 Washington Heights, Ann Arbor, MI 48109-2029, USA; berrocal@umich.edu; 6 Department of Epidemiology, University of Michigan School of Public Health, 1415 Washington Heights, Ann Arbor, MI 48109-2029, USA; marieo@umich.edu (M.S.O.); gronlund@umich.edu (C.J.G.); 7 NCAR JNT RAL, 3450 Mitchell Lane, Boulder, CO 80301, USA; jvigh@ucar.edu; 8 NESII—NOAA/ESRL, 325 Broadway, Boulder, CO 80305-3328, USA; luca.cinquini@jpl.nasa.gov

**Keywords:** foodborne disease, *Salmonella* infections, evaluation, temperature-based heat indices, ARRM and BCCA statistical downscaling methods, Washington D.C.

## Abstract

Foodborne diseases have large economic and societal impacts worldwide. To evaluate how the risks of foodborne diseases might change in response to climate change, credible and usable climate information tailored to the specific application question is needed. Global Climate Model (GCM) data generally need to, both, be downscaled to the scales of the application to be usable, and represent, well, the key characteristics that inflict health impacts. This study presents an evaluation of temperature-based heat indices for the Washington D.C. area derived from statistically downscaled GCM simulations for 1971–2000—a necessary step in establishing the credibility of these data. The indices approximate high weekly mean temperatures linked previously to occurrences of *Salmonella* infections. Due to bias-correction, included in the Asynchronous Regional Regression Model (ARRM) and the Bias Correction Constructed Analogs (BCCA) downscaling methods, the observed 30-year means of the heat indices were reproduced reasonably well. In April and May, however, some of the statistically downscaled data misrepresent the increase in the number of hot days towards the summer months. This study demonstrates the dependence of the outcomes to the selection of downscaled climate data and the potential for misinterpretation of future estimates of *Salmonella* infections.

## 1. Introduction

Temperature and precipitation extremes contribute directly and indirectly to disease incidence and impact human morbidity and mortality [1,2]. Higher temperatures have been linked to changes in occurrences of foodborne illnesses in many countries (Pacific Islands [3]; Peru [4,5]; England and Wales [6,7,8]; ten European countries [9]; Canada [10]; Australia [11,12,13,14]; China [15]; U.S. [16]; New Zealand [17]; northwest Russia [18]). Specific causative pathogens identified included *Salmonella* [8,9,10,11,12,13,14,16,18], *Cryptosporidium* [16,17,19], and *Campylobacter* [7,8,10,16]. Focusing specifically on *Salmonella*, research has demonstrated that the rate of growth of *Salmonella* sp. is associated with temperatures within the range 7.5 °C–37 °C [20], and *in vitro* studies of bacterial growth indicated faster hourly growth rates at persistently higher temperatures, e.g., 37 *vs.* 25 °C [21]. Although extreme precipitation events also contribute to the incidence of foodborne diseases, associations are not as strong as for temperature, and the direction of associations varies by location or time of year [13,14,18,22]. In this paper, we will focus on downscaled temperature data as they relate to occurrences of *Salmonella*.

Nowadays, the societal burden of foodborne infectious diseases is significant in industrialized and less-industrialized nations. For example, the U.S. Centers for Disease Control and Prevention (CDC) estimate that annually about 48 million people are sickened in the U.S., some 128,000 become hospitalized, and about 3000 die of foodborne diseases [23]. The economic impacts of foodborne diseases in the U.S., alone, are estimated to about $77.7 billion [24].

Projecting how the burden of foodborne diseases could change with climate change requires relevant climate information at the appropriate spatial and temporal scales. Further, the sources of information should be legitimate and the procedures and methods applied to develop these data should be transparent to the non-specialist [25]. Challenges with using Global Climate Model (GCM), also known as General Circulation Model, based projections include their large spatial scales. Finer-scaled climate information would be helpful for designing effective and efficient adaptation policies and programs to address projected changes in risk [1].

To address the questions of scale, the climate-science community is developing approaches to downscale GCM outputs to local and regional scales. Many data portals now provide easy access to downscaled climate model data at regional and local scales (e.g., [26,27,28,29]). These downscaled projections address the issues of mismatch between the spatial scales of available global climate model data and the information needed for evaluation of potential local impacts.

The salience of the temporal scales of climate data to *Salmonella*-related analyses requires consideration of the characteristics of *Salmonella* occurrences. Summarizing the epidemiological literature, no single health impact function (disease incidence per unit increase in temperature) is widely used to express the relationship between temperature and *Salmonella* infections. Research links the average weekly maximum or minimum temperatures to weekly occurrence of *Salmonella* infections, with lags of 2–5 weeks. At a finer temporal scale, Naumova *et al.* [16] identified a relationship between *Salmonella* infections and ambient temperature, where the peak of disease followed the peak in daily temperature with a lag of 2–14 days. Naumova *et al.* [16] note that daily time series data allowed the detection of seasonal peaks that would have been missed using weekly or monthly data.

More broadly, conclusions from the epidemiological literature review include that the relationship between temperature and *Salmonella* is affected by public health awareness and prevention practices. Foodborne illness incidences are not reduced due to acclimatization to heat in the way that heatstroke and heat-associated cardiovascular or respiratory risks might be reduced due to acclimation [30,31]. Epidemiological models suggest that reliance on mean temperatures on time scales longer than even one week may obscure important relationships between temperature and occurrence of illness. Therefore, the relevance of climate-model projections to *Salmonella* benefits from the consideration of variability on daily to weekly time scales. Finally, the absolute rather than the percentile temperature measure is likely more important for studies focused on foodborne illness risks.

Statistical methods are often used to evaluate the spatial and temporal characteristics of climate-model simulations. A standard evaluation technique is to use simulations of past (historical) periods for comparisons to observations to determine the skill of the simulations. Considering the results of epidemiological studies that link weekly and sub-weekly temperature variability with an adverse health outcome brings physically based conditionality to statistical evaluations. In the atmosphere, temperature variability on time-scales of 1–3 weeks is linked to specific meteorological processes. For example, persistent warm temperatures, lasting for several days and leading to higher average weekly temperatures, are associated with meteorological features such as anticyclones or blocking events (e.g., [32,33,34,35]). Blocking describes a pattern of atmospheric flow that slows or stops the normal movement of storms from west to east around the Earth. Blocking is both geographically and seasonally dependent [36], and difficult to simulate in climate models as well as difficult to predict with weather models. Due to these meteorological processes, warm-air outbreaks occur in distinct episodes at specific locations, rather than being spatially and temporally random. Therefore, the evaluation of the appropriateness and robustness of climate model information in relation to *Salmonella* occurrences benefits from both statistical and physical process considerations.

The need for both evaluation of the statistical and the physical characteristics of models suggests that health impacts researchers and climate scientists need to work together in order to establish the salience of climate-model data to *Salmonella* impacts. This conclusion is consistent with that of Dilling and Lemos [37] that “nearly every case of successful use of climate knowledge involved some kind of iteration between knowledge producers and users”. In other words, use of climate-model data requires guidance on “how to choose an appropriate data set, assess its credibility, and use it wisely” [38]. Such guidance should be based on a thorough and comparative evaluation of the historical characteristics of the available datasets not only in terms of their capacity to represent the mean state of the local climate but also to capture the variability related to meteorological processes.

In this paper, a team of climate scientists, epidemiologists, and information technology experts evaluate the supply chain of climate information from model simulations through bias correction to downscaling. That is, the evaluation is framed by the epidemiological application. The climate-model data that are used come from products proffered by climate scientists as useful, and we investigate their usability for this specific application.

We consider temperature-based heat indices obtained from GCM outputs and two widely available to practitioners statistical downscaling products. The heat indices were derived for five areas including Wayne County, Michigan (MI); South-East MI climate division; Cuyahoga County Ohio (OH); North-East OH climate division and the Washington, District of Columbia (D.C.) area. These areas were chosen because of long-standing research assessing and modeling population vulnerability to climate change in these cities. Baseline thermal characteristics using the temperature-based heat indices were established for all five areas. For one area, Washington, D.C., we analyze, in detail, how the heat indices’ representation changed when coarse GCM data were transformed by re-gridding, subsequent bias-correction and downscaling. The paper is organized as follows: Section 2 describes the data and the methodology used in the study, Section 3 illustrates and discusses the results, while Section 4 summarizes our conclusions.

## 2. Experimental Section

### 2.1. Study Details

Based on the literature review, we chose three indices of high temperature, HD30, HD35, TR (Table 1). They are prominent in the existing climate impacts literature and CDC has used HD35 to project potential future climate impacts on human health [23]. The chosen heat indices do not match exactly empirically derived temperature thresholds that could be linked to increase in *Salmonella* occurrences in our areas of interest, however, HD30, which is the main focus of the analyses, represents hot days with maximum temperatures high enough to promote faster growth rates of foodborne pathogens. In addition, such high temperatures are often experienced in our study locations. HD35 and TR will be used primarily to establish the climatic background characteristics of the areas of interest.

We evaluated the time series for HD30, HD35 and TR for the months April to September over a World Meteorological Organization (WMO) climate normal period, 1971–2000. By comparing the indices from observations with climate model-based data, we determine how closely the model-derived indices represent the thermal conditions during the historical period. A good representation of the observed heat indices increases our confidence in the ability of the downscaled data to represent the thermal conditions, and hence, the potential for *Salmonella* occurrences in historical (and possibly future) periods.

To test whether the temperature indices are appropriate proxies for high weekly mean temperatures, associated with *Salmonella* infections, we calculated the correlation between weekly HD30 and average weekly tasmax for the months April-September, and for the summer months, June-August, only. For the 30-year analysis period, we used the 2°-latitude re-gridded observed data of Maurer *et al.* [39], (Maurer02v1_2deg, see Table 2), used subsequently in our analyses, for nine grid cells overlaying a large region that encompasses Washington D.C. For June-August (April-September), the correlations, as indicated by the Kendall tau rank correlation test [40], were significant and varied between 0.53 (0.44) and 0.81 (0.81) over the nine grid cells, with 0.81 (0.77), for the grid-cell covering, just, Washington, D.C. The Kendall tau rank test was chosen because, as a non-parametric test, it is robust to non-normality of the data. The results of this test increase our confidence that the indices were appropriate for the goals of this study.

**Figure 1:**
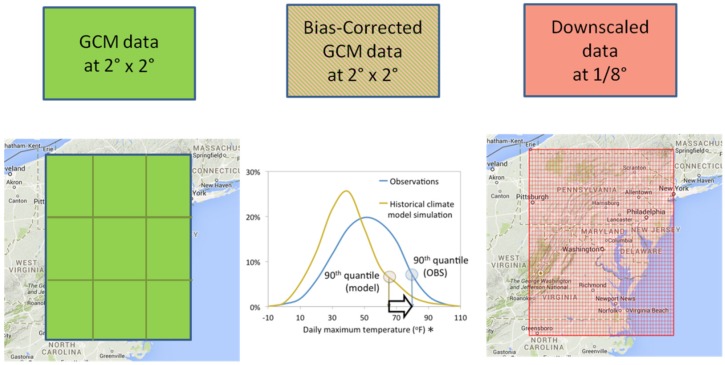
Chain of BCCA based temperature projections used in the study.Notes: * source is adapted from [41].

We also investigated how the tailoring performed through a three-stage downscaling process influenced the uncertainty characterization for the BCCA downscaled data. The chain of processes includes: (1) GCM datasets re-gridded to 2° latitude resolution; (2) bias-corrected (BC) 2° latitude resolution GCM data; and (3) fully downscaled GCM (Figure 1).

### 2.2. Details about the Datasets

#### 2.2.1. Downscaled Data

The heat indices (HD30, HD35 and TR) were calculated from daily tasmax and tasmin data, obtained from two widely-available to scientists and practitioners statistical downscaling products developed by applying the Asynchronous Regional Regression Model (ARRM, [42]) and the Bias Correction Constructed Analogs (BCCA, [43]) method. We used the ARRM and BCCA downscaled products available for 8 GCM simulation suites that are part of the World Climate Research Programme's (WCRP's) Coupled Model Intercomparison Project phase 3 (CMIP3) multi-model dataset [44]. The downscaled datasets provide daily output for the conterminous US at an approximately 12 km spatial resolution (Table 2) and we calculated the heat indices at each grid cell on a monthly scale.

#### 2.2.2. Baseline Observed Data

The downscaled data were compared to indices calculated from the observation-based gridded dataset of Maurer *et al.* [39] version 2 with the same 12 km resolution (*Maurer02v2_1/8*, Table 3). To determine the bias correction for the BCCA and the ARRM methods, an older version of this dataset was used by the downscalers, namely Maurer02v1. Although two different versions of the Maurer02 data are used for bias-correction and for comparison to observations, the National Climate Predictions and Projections (NCPP) project in evaluating differences between the two versions concluded that “Most users could consider the v2 to be a simple extension of v1, except in the special cases we have identified.” (https://earthsystemcog.org/projects/obs_evaluation/evaluationReport). None of these special cases, related primarily to topography near regionally large watershed boundaries, were identified in the areas of interest to this study.

#### 2.2.3. Global Climate Model Data

The eight GCMs used in the analyses are listed in Table 4; the GCMs were chosen based on the availability of tasmax and tasmin data downscaled using both statistical downscaling methods. Temperature data from the 20th century simulations (20C3M) were used. The eight GCMs adequately represent the historical climate on large (e.g., the Tropics, or the extra-tropical zones in the Northern and Southern Hemispheres) and/or global scales [45,46].

To complete our investigation of all the steps in Figure 1, *i.e.*, the GCM re-gridded and bias-corrected data, produced by sequential steps in the BCCA method, GCM tasmax time series for the same 8 GCMs, re-gridded to 2° latitude × 2° longitude common resolution (*GCM_2deg*, Table 2), were obtained from the “Downscaled CMIP3 and CMIP5 Climate and Hydrology Projections” archive [47,48]. These data were the basis for the calculation of the re-gridded (*GCM_2deg*) and re-gridded and bias-corrected GCM (*BC GCM_2deg*) temperature indices. The climate model-based indices were compared to the Maurer *et al.* [39] v.1 tasmax data (*Maurer02v1_2deg*) at 2°latitude × 2°longitude resolution at nine grid cells, centered on 39°N and −77°E, which overlap with the Washington D.C. and surrounding areas. The observed, re-gridded GCM and bias-corrected GCM time series with coarse resolution were compared grid cell by grid cell.

### 2.3. Geographical Areas of Investigation

The downscaled indices were calculated for each grid-cell at 1/8° resolution within the five areas of interest—Wayne County MI; South-East MI climate division; Cuyahoga County OH; North-East OH climate division and the Washington D.C. area. The Washington D.C. area includes Washington D.C. and the following counties: Montgomery and Prince George’s in Maryland, and Alexandria, Arlington, and Fairfax, in Virginia. The county scale is a standard widely used in human health impacts literature. We also included climate divisions [49] as aggregation units, since the National Oceanic and Atmospheric Administration (NOAA) provides quality-controlled products and summaries at the climate-division scale and applied-climate scientists often use them. The climate division areas in this study encompass the counties of interest and were used to perform sensitivity analyses related to the spatial scale of the calculated indices. The Washington D.C. area is a special case and does not have a single corresponding climate division.

### 2.4. Calculation Details and Summary Statistics

#### 2.4.1. Baseline Characteristics of the Monthly Temperature Based Heat Indices for the Areas of Interest

The baseline thermal characteristics of the five areas were investigated using the monthly time series of HD30, HD35 and TR from the *ARRM_ensemble_1/8* and the *BCCA_ensemble_1/8* and the observational data (*Maurer02v2_1/8*). The indices for the five geographical areas were extracted from a nation-wide grid of calculated monthly grid cell indices using the Open Climate GIS (OCGIS) tool [50] and a “clip” operation (http://ncpp.github.io/ocgis/appendix.html#clip-intersection). Using the monthly heat indices (number of hot days or nights per month) for every year obtained at each grid cell within the five areas we calculated grid cell 30-year monthly climatologies (period mean values for 1971–2000). Absolute grid cell-specific biases were defined as the difference between these 30-year climatological monthly means of the downscaled and the corresponding observed indices. To obtain areal means that correspond to the non-regular counties or climate divisions, we averaged spatially the grid cell 30-year monthly mean values of the indices and the absolute biases for the grid cells lying within the areas of interest using an area-weighting method. Percent biases were calculated subsequently using the *Maurer02v2_1/8* based areal mean values of the indices as the baseline. Finally, using R version 3.2.1. [51], we generated box plots for the areal mean values and biases of the period mean of the indices.

#### 2.4.2. Frequency of Hot Days (HD30)

We compared the 30-year monthly time series of HD30 within the Washington D.C. area using both the ARRM and BCCA downscaled output (*ARRM_ and BCCA_ensemble_1/8*) and the *Maurer02v2_1/8* observed data at 12 km resolution. Using the same area-weighting procedure as above, grid cell HD30 values were aggregated to generate an areal monthly mean for every year, thus resulting in time series of areal monthly mean HD30 values.

The HD30 areal mean time series from the *ARRM_* and *BCCA_ensemble_1/8* data, from the *Maurer02v2_1/8* observed data as well as the *GCM_2deg*, *BC GCM_2deg* and *Maurer02v1_2deg* HD30 time series from the grid cell overlapping the Washington D.C. area were used to plot monthly histograms of the number of hot days (HD30) for the 1971–2000 period. The number of bins for the histograms were a compromise between the number of bins determined using the formula suggested by Panofsky and Brier [52], where the number of classes is equal to 5log_10_(n), with *n* being the number of observations, and the desire to have equally sized bins. The histograms represent the relative frequency of the years with a given HD30 (frequency of years with hot days/total number of years) for each bin.

Finally, we used the Brunner-Munzel test for stochastic equality of two samples [53], available in R (package *lawstat*), to investigate the statistical significance of the differences between the monthly distributions of the HD30 index obtained from downscaled, 2° bias-corrected, and the re-gridded to 2° GCM data in comparison to the respective observational dataset. The Brunner-Munzel (B-M) test is robust against outliers, does not assume that the underlying distribution functions are continuous and as a consequence it can perform well with ties in the data.

### 2.5. Description of the Statistical Downscaling Methods

The ARRM method [42] downscales the large-scale temperature output from a GCM to approximately 12 km resolution, or 1/8°, by fitting a piecewise linear regression that relates observed and modeled quantiles of daily temperature. To avoid obtaining unrealistic values at the extreme tails of the distribution, an additional post-processing step is included, which consists in further bias-correcting the extreme quantiles below the 5th and above 95th percentile to match the observations. The ARRM method is applied to daily data separately for each month to allow for a better intra- and inter-annual representation of the observed variables. The method has been evaluated and found to be “efficient and robust” [42]. Downscaled temperatures between the 0.1th and 99.9th percentiles across the United States exhibit biases of less than 1 °C compared to observations [42].

The BCCA method [43] is based on an explicit bias-correction (BC) step of the entire distribution, where the modeled quantiles of the specific variable distribution are mapped onto the observed quantiles with the GCM data re-gridded and the observed data aggregated to 2° resolution. This step, called “quantile mapping”, is applied on the daily values for a given variable, though each month is treated separately and the bias-correction is month specific. Although the purpose of the BCCA method is to match the distribution of the modeled variable to that of the observed variable, it does the fitting only over the calibration period. Therefore, this process still allows the mean and the variability of the GCM data to change in response to future changes in forcing conditions. The next step in the downscaling procedure for the BCCA method is the Constructed Analogs (CA) step, which is based on the idea that analogs for a GCM daily weather pattern can be constructed using a combination of weather patterns from several observed days [24]. During this step the sequencing of the daily weather events follows that of the coarse resolution GCM input [43] and the skill in representing the daily variability of a variable depends on the GCM skill. In a climate change context, the projected changes in temperature will depend on the changes in the frequency and amplitudes of the daily weather patterns [54]. The BCCA method has been found to skillfully reproduce observationally driven stream flows in the U.S. [43].

Because the downscaling methods were not designed to capture or correct for temporal autocorrelation (sequencing) of weather events, the ability of the ARRM and BCCA downscaled data to reproduce the temporal autocorrelation in the observed extreme weather events depends on the skill of the GCMs to reproduce daily variability. Finally, statistical downscaling methods rely on the assumption that the statistical relationship they are based on will remain valid in future climate conditions. The impact of this assumption is a subject of ongoing research [41,55].

## 3. Results and Discussion

### 3.1. Observed Thermal Characteristics Based on Maurer02v2 Data

**Figure 2:**
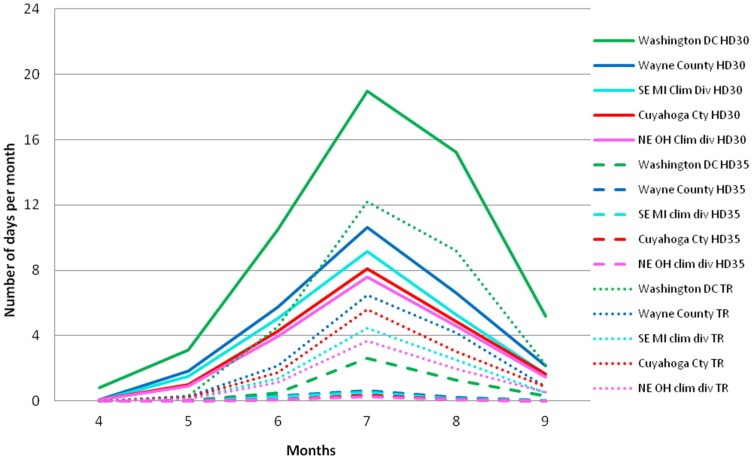
Monthly distribution of the average HD30, HD35 and TR for the period 1971–2000 for all areas, derived using the Maurer02v2 observational dataset.

Monthly 1971–2000 means of HD30, HD35, and TR trace the annual pattern of temperature (Figure 2). There is a peak in July for all indices. For all areas, HD30 and TR are mostly zero in April while only occasionally register events in May (on average less than 5 hot days or warm nights per year) similarly to the number of hot days and nights in September. The thermal characteristics represented by the indices in June are similar to those in August. Hot days with tasmax above 35 °C were uncommon. In general, the Washington DC area experienced a greater number of hot days and nights compared to the northern areas. The MI areas had higher monthly HD30 and TR on average for the 30-year period compared to the respective OH county and climate division areas.

In the sensitivity analysis comparing the heat indices results aggregated over climate divisions as opposed to counties, we found minimal differences between the county and climate division results for all indices (Supplementary Material Table S1). Therefore use of products at the scale of climate divisions is justified for applications at the county scale for our areas of interest, and we will present no more climate division results.

### 3.2. Comparison of the Period Mean of the Indices Calculated from Observed Data and the ARRM and BCCA Downscaled Ensembles

**Figure 3:**
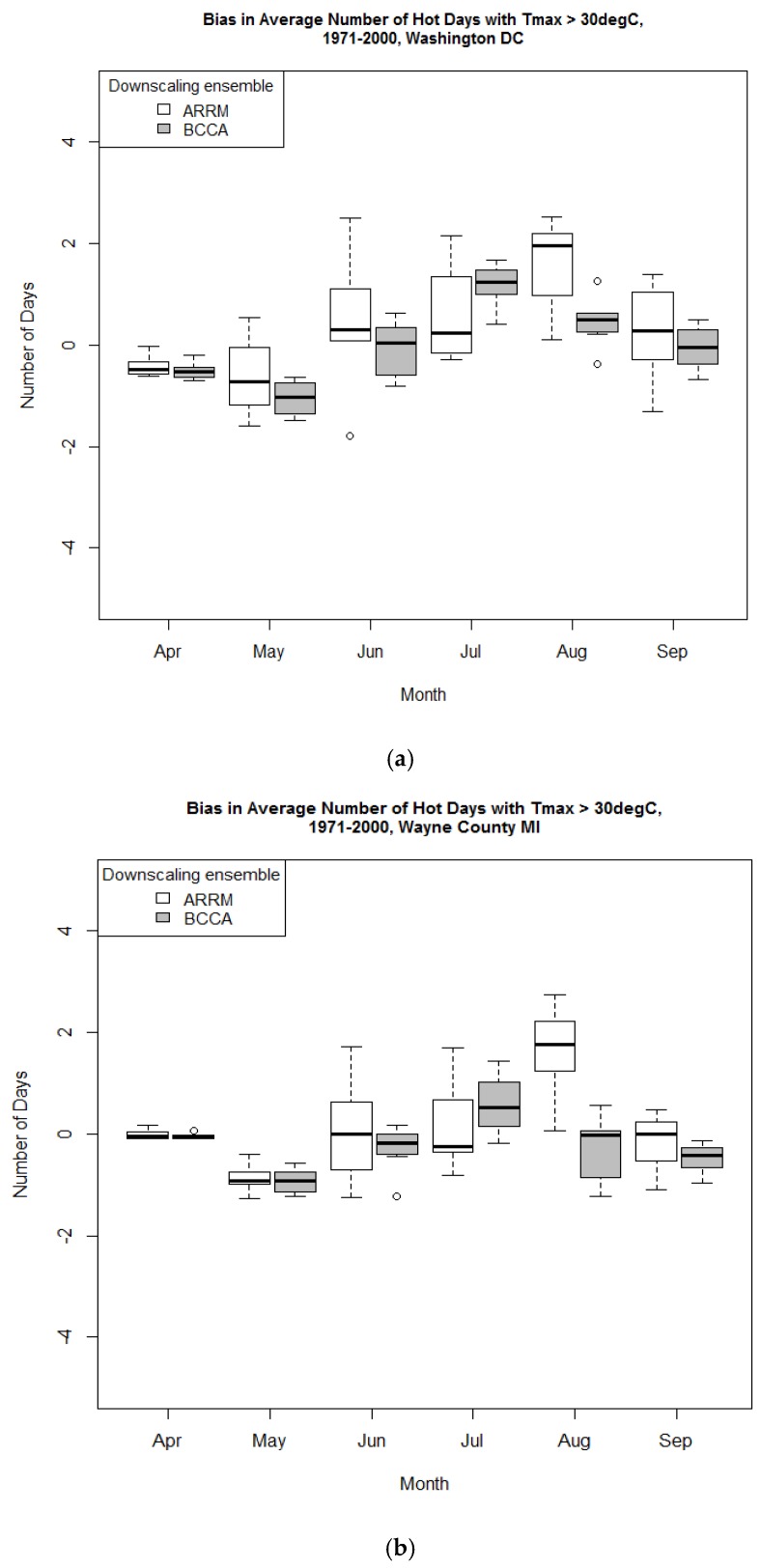
Absolute bias relative to Maurer02v2_1/8 observational dataset of the period mean HD30 in (**a**) Washington DC area and (**b**) Wayne County MI for 1971–2000 as represented by the ARRM and BCCA ensembles at 1/8° latitude resolution. Heavy line within each box plot represents the median.

**Figure 4:**
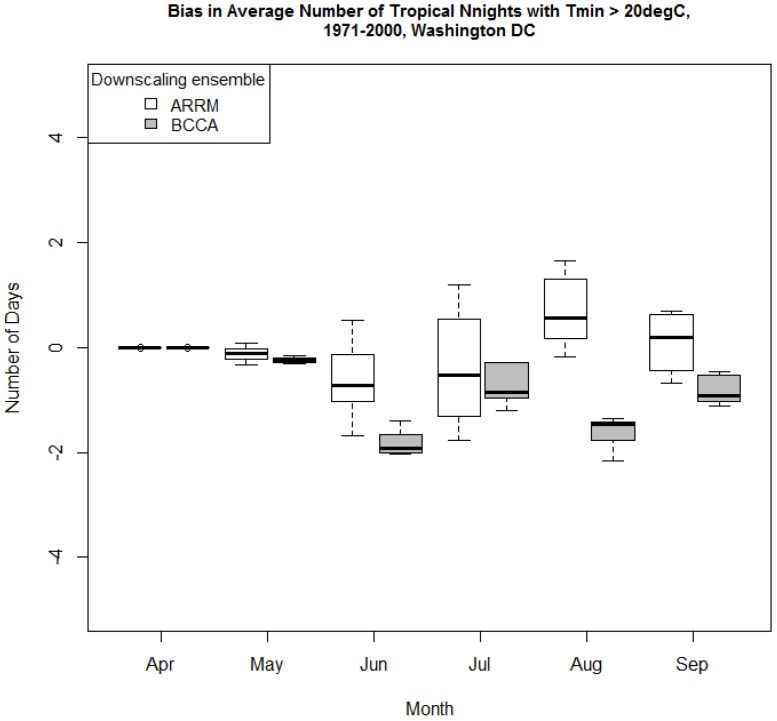
Absolute bias relative to Maurer02v2_1/8 observational dataset of the period mean number of tropical nights for Washington D.C., 1971–2000, as represented by the ARRM and BCCA downscaled GCM ensembles at 1/8° latitude resolution. Heavy line within each box plot represents the median.

**Figure 5:**
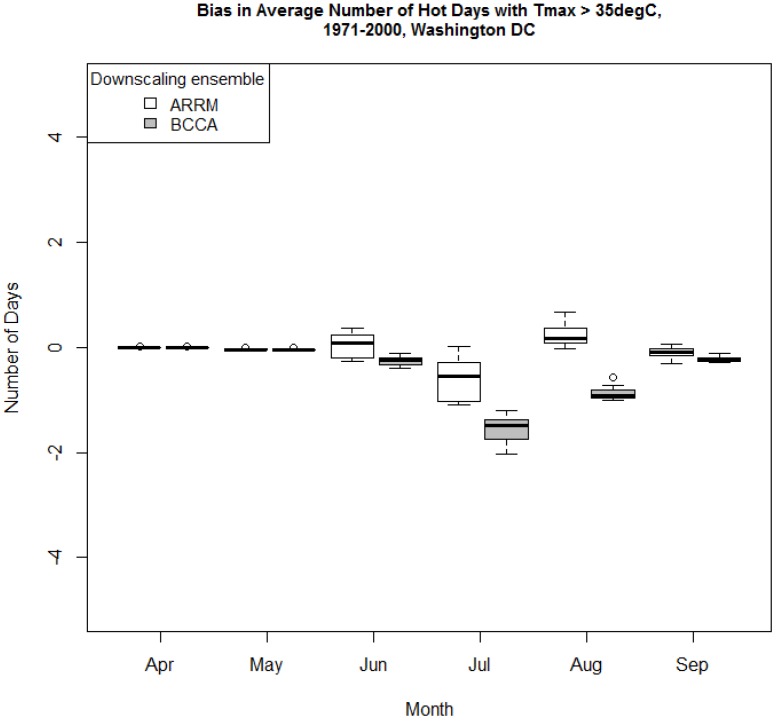
Absolute bias relative to Maurer02v2_1/8 observational dataset of the period mean HD35 for Washington DC for 1971–2000 as represented by the ARRM and BCCA downscaled GCM ensembles. Heavy line within each box plot represents the median.

The number of hot days and hot nights represented by the three indices somewhat differed in the observed and downscaled data. Figure 3, Figure 4 and Figure 5 present the biases relative to the *Maurer02v2_1/8* observational dataset which are discussed here in terms of the median of the *ARRM_ and BCCA_ensemble_1/8*. The absolute biases are generally small for the period mean number of hot days and nights for all areas and all indices, as expected, because the GCM data have been bias corrected. The HD30 absolute bias varies generally between 0 and up to about 2 days in August. During August, the ensemble median of the ARRM downscaled data overestimates HD30 in all areas (Figure 3 and Supplementary Material Table S1).

For example, in comparison to the observed values, the ARRM ensemble median overestimates HD30 by about 13% in Washington D.C. and by about 22% to 35% in the northern areas (lower bias values for the MI areas compared to the OH areas). In comparison to the observed values, the BCCA ensemble median overestimates HD30 by about 3% in August in Washington D.C. and underestimates HD30 by about −3% to −10% in the northern areas. In general, the median of the BCCA-downscaled GCMs underestimates HD30 for the rest of the months. For the early months of the warm period the BCCA and the ARRM downscaled data generally underestimate the observed HD30, based on the median of the ensemble results. Since the observed HD30 are rare in the beginning of the warm season, the percent biases between the downscaled and observed indices are often larger for April and May.

The absolute biases for TR are, likewise, small and vary between 0 and 1 day for the northern areas and up to 2 days in Washington D.C. (Figure 4, Washington D.C. only). These small biases equate to about 6% to −16% (sign is based on the downscaled data used, ARRM and BCCA, respectively) in August for Washington D.C. and about 20% to −25% for the northern areas for the same month. In general, TR is underestimated by BCCA and ARRM for almost all months. Since the observed frequency of these tropical nights is low at the beginning of summer, the relative biases are somewhat larger. For example, in Cuyahoga County, the percent bias is −23.5% (for BCCA, Supplementary Material Table S1) in June compared to −16.7% in August for TR estimates.

For the northern areas, HD35 are rare in both the observations and the downscaled data. The HD35 for the Washington D.C. area (Figure 5) are underestimated (up to 1.5 days of absolute bias) in July and in August by the BCCA ensemble. However, this corresponds to a relative underestimation of between 54% and 70%.

These bias-corrected, downscaled data were designed to represent the primary variable, temperature, well. This analysis shows that there are some small differences when heat indices are calculated. HD35, our highest temperature index, and TR are slightly underestimated in the summer, June, July, August, but the differences are generally minor and may not be statistically significant. Outside of these months, these extremes are rarely observed. The more moderate heat index, HD30, shows an underestimate in May and over- or under-estimate in July and August, depending on the downscaled dataset. During August, the ARRM ensemble median based biases are greater than those based on the BCCA ensemble median in all the areas analyzed. These differences were not evaluated for statistical significance.

Overall, the downscaling methods represent well the period means of climate data (as indicated in the literature), and even of indices such as HD30, HD35 and TR, as indicated by our analyses. The representation of the period mean of the HD30 and the rest of the indices relates to the abilities of the downscaling methods to correct for GCM biases in the mean and in the quantiles of the tasmax and tasmin monthly distributions compared to observations.

We continue the more detailed investigation of the characteristics of the downscaled datasets and their potential impacts on the occurrence of *Salmonella* infections only for Washington D.C. Hot days with tasmax above 30 °C occurred frequently enough in Washington D.C. to allow for insightful comparisons among the downscaling methods.

### 3.3. Analyses of the Representation of the Frequency of High Temperatures by Observed and Downscaled Data and Relation to Salmonella Occurrences

HD30 was analyzed for the Washington D.C. area to look in greater detail at the actual distribution of the incidences of hot days. The goal was to evaluate the ability of the climate model-based data to represent the occurrence of hot days that are relevant to *Salmonella*. This brings attention also to the GCMs, themselves, because before bias-correction the actual number of hot days in any month, season and year depends on the sequences of daily weather patterns as simulated by the GCMs. We use histograms for HD30, based on the time series of the monthly area mean HD30 for the period, for each downscaling method and evaluate, statistically, any potential differences between the observed and the downscaled monthly distributions of HD30. Here in our discussion of the results, we focus, primarily, on the BCCA method because we have access to step-wise data from the procedural steps of the method outlined in Figure 1.

**Figure 6:**
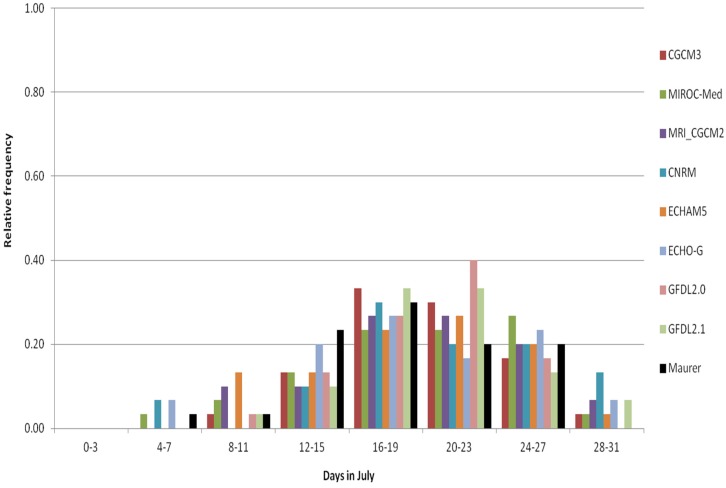
Histogram of HD30 in July, 1971–2000, Washington DC area, as represented by the Maurer02v2_1/8 observed data and the individual BCCA downscaled GCM time series (BCCA_ensemble_1/8).

We start our analysis with the last element of the climate information chain (Figure 1) by comparing the statistically downscaled CMIP3 GCM data to observations, the natural starting point of analysis for practitioner’s impact studies. In Figure 6 (for the ARRM method, see Supplementary material Figure S1) the observation-based HD30 for July is represented in black. The observed data distribution is relatively well represented by the downscaled GCM data using the BCCA and the ARRM downscaling methods. The downscaled data for some models feature years with more than 28 days with high tasmax within July, which is not observed in the 1971–2000 period. Compared to the BCCA downscaling, the ARRM downscaling enhances both extremes, especially the higher extremes, *i.e.*, the ARRM downscaled GCM data show somewhat larger number of years in the higher end of the distribution compared to the BCCA downscaled data. This difference between the ARRM and BCCA downscaled GCMs and the observed distributions is generally evident from June to September (Supplementary Material Figures S2–S4).

Though there are some qualitative differences at the extremes, as indicated by Figure 6, statistical analysis using the Brunner-Munzel test indicates that there are no statistically significant differences for years with HD30 derived from the observed and the downscaled GCM data, with the exception of: (a) for BCCA—the CNRM downscaled data for April, the GFDL21 downscaled data for April and May, as well as the MIROCmed downscaled data for May; and (b) for ARRM—the CNRM downscaled data for April, May and September; the ECHO-G, MIROCmed and MRI-CGCM2 downscaled data for April; the GFDL21 downscaled data for May (Supplementary Material Table S2).

For the early months of the warm season, April and especially in May, some of the downscaled GCMs, specifically those characterized by statistically significant differences from the observations, have quite a few years with no hot days (HD30), while the observations show the regular occurrence of such hot days (Supplementary Material Figures S5 and S6). The bias-corrected, downscaled data from the significantly different models underestimate early season heat, as represented by the higher bins of the observed distribution in April and May, and do not capture well the tasmax transition from spring to summer (Supplementary Material Figures S5 and S6).

**Figure 7:**
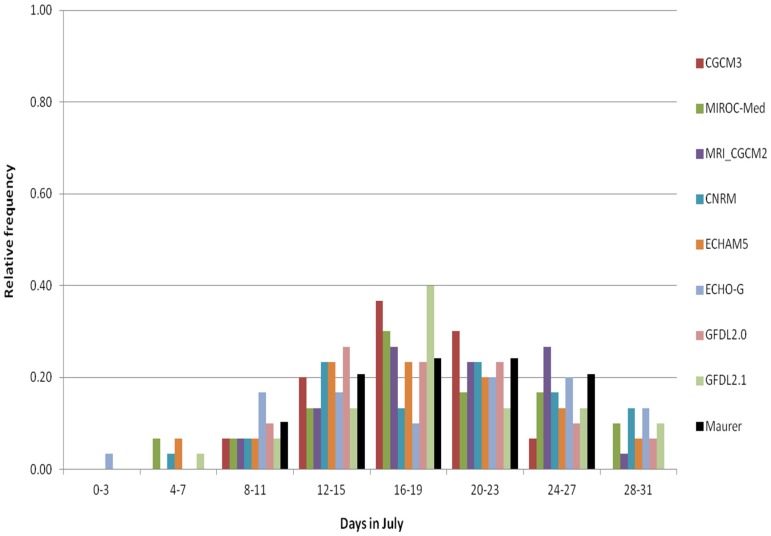
Histogram of HD30 in July for a grid cell that overlays the Washington DC area, based on the bias-corrected individual 8 CMIP3 GCMs re-gridded to 2° × 2° resolution—BC GCM_2deg data, 1971–2000.

**Figure 8:**
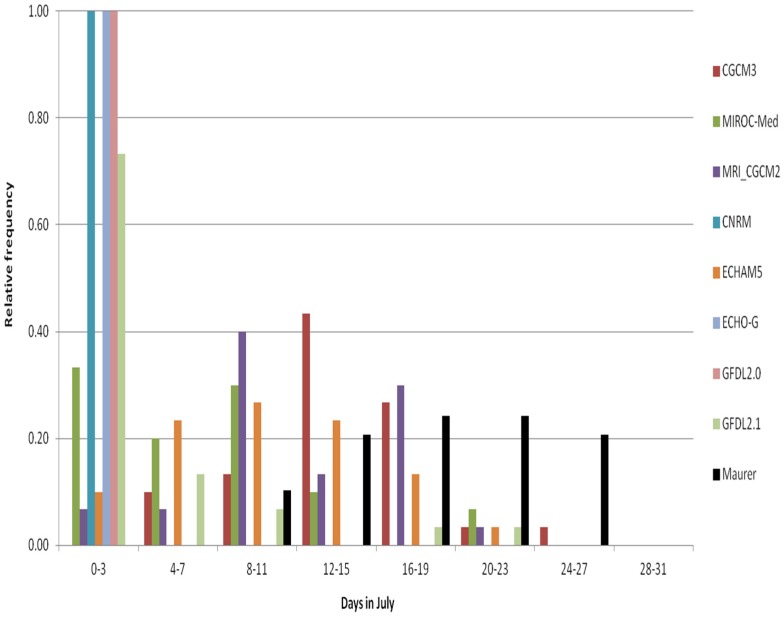
Histogram of HD30 in July for a grid cell that overlays the Washington DC area, based on the individual 8 CMIP3 GCMs re-gridded to 2° × 2° resolution—GCM_2deg data, 1971–2000.

We will continue our investigation of the climate information chain specifically for the BCCA method (Figure 1) to describe the relationship between the downscaled data and the information that the GCMs provide. Figure 7 and Figure 8 show the GCM results, with no downscaling, re-gridded to a common 2° latitude resolution. The data in Figure 7 (*BC GCM_2deg*) have been bias corrected using a quantile mapping procedure and represent an intermediate step of the BCCA downscaling method. In Figure 8 the GCM data have no bias-correction (GCM_2deg), but also represent a step of interpolation to common grids in the BCCA downscaling method. The following discussion focuses on the impacts of these procedures in relation to the BCCA downscaling method.

The bias-corrected CMIP3 models produce a wide distribution (Figure 7). Similar to the bias-corrected BCCA downscaled GCM data, the bias-corrected CMIP3 models extend beyond the lower and the upper range of the observations. Some of the models depict years with less than 8 or more than 27 hot days. Despite the extended range, none of the 2-degree re-gridded and bias-corrected GCM indices were statistically different from the 2-degree observationally based HD30 indices (Supplementary Material Table S2). As seen from the discussion of the downscaled indices, however, the subsequent step after the bias-correction in the BCCA downscaling method introduces additional uncertainty for some of the GCM downscaled data, *i.e.*, the statistically significant exceptions for April and May mentioned above, thus highlighting issues related to the ability of some of these downscaled data to represent accurately early season hot days occurrences.

For the re-gridded GCM data without bias correction (*GCM_2deg*), three of the models show ***no*** hot days above 30 °C in July for any year (Figure 8). Without bias correction, the CMIP3 models have a distinct cold bias (fewer occurrences of hot days above 30 °C throughout the years) compared to the observations for Washington D.C. in all months (Supplementary Material Figures S7–S9). No models represent the highest bins well. For the Washington D.C. area, the bias correction is found to be quite large, and accounting for early as well as mid-season hot days.

In addition to potential shortcomings in the representation of processes important to local weather and climate in the GCMs, there are other possible sources of uncertainty related to post processing and tailoring of the 2-degree re-gridded model simulations that could bring about such distinct cold bias as the lack of hot days above 30 °C. We note, specifically, sensitivity to the land-mask details, which are used to distinguish land from water. Land and water have different thermal characteristics and, during the warm season, over-representation of the influence of water would lead to a cold bias. Specifically for our area, the re-gridding step, in combination with the atmosphere horizontal resolution for some models being coarse (for example, for ECHO-G, see Table 4), may lead to inclusion of information predominantly from ocean grid cells and hence may contribute to depicting cooler temperatures.

The results from the Brunner-Munzel test confirm that the differences between the 2-degree observed and the 2-degree GCM re-gridded HD30 monthly distributions are statistically significant at 5% significance level in almost all cases (Supplementary Material Table S2). In a few cases for July and August the test is not able to present results other than NA (Not Applicable) for three of the GCMs (CNRM, ECHO-G and GFLD20). The contrast between the lack of HD30 for these three re-gridded models compared to the observed data, which do indicate a large number of days with high maximum temperatures throughout the years, is most striking during these two months, leading to the test not performing well under these circumstances.

### 3.4. Association of Salmonella Occurrences with High Temperatures

Epidemiological literature indicates that a set of high-temperature days in a weekly timespan is important to the occurrence of *Salmonella* infections. We used the number of days in a month with maximum temperatures above a high (30 °C) threshold (HD30) as a proxy for high weekly temperatures. Multiple hot days or persistent periods with high temperature in a month will relate closely to high weekly means; hence, should be relevant to the incidence of *Salmonella*. The analysis, thus far, evaluated the ability of two, widely used, downscaled datasets to represent temperature-based heat indices. We deconstructed the information chain (Figure 1), to understand the impacts on data and information of major tailoring steps of the BCCA downscaling method applied to climate information from GCMs for use by practitioners. We considered *Salmonella* infections, which are related to the frequent occurrence of warm, not necessarily the hottest, temperatures, over extended periods such as a week. Therefore, credible representation of both magnitude and variability of temperature is required.

Having in mind the basic positive relationship between high weekly mean temperatures and incidences of *Salmonella* infections, it is straightforward to conclude that, for our study area, the 2-degree re-gridded GCMs without bias correction would grossly under-represent the occurrence of *Salmonella* infections. With bias correction, the temperature range of the climate models overlap with observations as well as with the *Salmonella* infections impact function. Therefore, the bias-corrected GCMs are capable of representing a relation between temperature variability and *Salmonella* infections. Focusing on the bias-corrected, downscaled climate models, the products most tailored for use by practitioners, the downscaled models represent generally well the observed distribution of HD30. However, additional uncertainty introduced by the remaining step in the BCCA downscaling method is evident for some of the downscaled GCMs (CNRM, GFDL21, MIROCmed) for the early part of the warm season (April, May), highlighting the compound impacts of downscaling method and GCM capabilities to represent local climate on the credibility of the downscaled data. In comparison, the ARRM downscaled heat indices also exhibit statistically significant differences in their distributions for the early warm season for some of the GCMs (CNRM, ECHO-G, GFDL21, MIROCmed, MRI-CGCM2). In summary, some of the downscaled models misrepresent the observed distribution of HD30 in the early part of the warm season, and therefore may contribute to a different description of *Salmonella* infections occurrences during that period.

The bias correction of the GCMs contributes to a frequency distribution of temperature indices broader than observed, with higher and lower number of hot days. This wider range propagates through the information chain to the downscaled data. Though the occurrences of large and small numbers of hot days in a year are small enough that the bias corrected GCMs and the observations are statistically the same, the presence of these days contributes to the *Salmonella* uncertainty description. That is, when meteorological processes are considered the presence of extremes outside of the observed range warrants further consideration. *Salmonella* impacts and other applications that may be related to persistent or longer lasting weather patterns, such as considered here, present challenges to the downscaled products as well as the underlying GCMs. Although the statistical downscaling methods evaluated here were not explicitly developed to downscale heat indices or sequences of high temperatures, in general, they performed quite well as indicated by the statistical evaluation. The most prominent deficiency identified by this analysis is that early warm season is likely to be under-represented even by some of the downscaled data. It is not possible to generalize this result, however, to other geographic locations.

Given that the epidemiological studies reveal sensitivity to temperature that varies from study-to-study, perhaps from location-to-location, we can conclude that there are subtleties of the relation between *Salmonella* infections and environmental temperature. The shortcomings of some downscaled climate models in representing the HD30 variability on intra- and inter-annual time scales may be large enough and may overwhelm the subtleties of the *Salmonella* relation to temperature in early warm season. These considerations may require further and more detailed investigation of the representation of specific meteorological processes and underlying circulation patterns leading to occurrences of days with high maximum air temperatures (e.g., HD30) in the GCMs that exhibit statistically significant differences from the observational indices using data at the models' native resolution.

Bringing attention to the meteorological processes, especially blocking, May is a month with high number of blocking situations [36]. These circulation patterns persist for several days, often a week or longer. The occurrence and sequencing or persistence of such patterns and how well are they simulated depends on the underlying GCM that is being downscaled, specifically, its skill to represent the physical processes that bring about such hot days and its ability to depict the daily weather variability. The underrepresentation of early season hot day occurrences is consistent with potential underestimation of blocking by some models. This would not be corrected by either the bias correction or the rest of the downscaling steps in the methods used here. This is an example of where efforts to improve the ability of climate models to represent these weather-scale processes would have potential influence on indices related to important societal impacts, *i.e.*, *Salmonella* risk.

## 4. Conclusions

This study investigated the potential impacts of employing two widely available downscaled datasets to project how future changes in hot days might affect the occurrences of *Salmonella* infections. It also contrasted the high-resolution results with the underlying GCM output that is available at much coarser resolution for one of the downscaling methods. We used the indices of HD30, HD35 and TR, as proxies for high weekly mean temperatures, which have been found to lead to occurrences of *Salmonella* infections. To investigate the potential for credible representation of the future occurrences of *Salmonella* infections using temperature projections, we evaluated the abilities of the downscaled datasets to represent the temperature characteristics during the historical period, 1971–2000.

The evaluation of the representation of the period means of the indices demonstrated that:

- The ARRM and BCCA downscaling methods represented the period means of the number of hot days and hot nights for 1971–2000 well overall. This is expected since these downscaling methods are developed to adjust and bias-correct the means and the quantiles of the GCM data distributions to match the observed quantiles of daily tasmax or tasmin, in our case.
- The greatest differences between ARRM and BCCA are found in the peak of the summer season, July and August, when ARRM overestimated and BCCA often underestimated the observed 30-year period means of some indices, however, these differences are not evaluated for statistical significance.
- There is minimal difference in the results when counties or climate divisions, standard aggregation units of several counties, are used in our analyses.

Looking at the frequency distribution of HD30 the results indicated that, overall, the monthly observed distributions of HD30 are represented well by the statistically bias-corrected and downscaled GCM data with a few exceptions. Although the bias-correction procedure remedies the differences between the observed and the 2-degree re-gridded GCMs, the final step in the BCCA downscaling method does contribute additional uncertainty for some of the downscaled GCMs in April and May. Furthermore, the ARRM downscaled data also do not represent well the occurrence of hot days in the early warm season for some GCMs as indicated by the statistical evaluation. More specifically:

- In April and May some of the CMIP3 bias-corrected and downscaled using both downscaling methods GCMs miss the increase in HD30 towards the summer months.
- The application of the BCCA complete downscaling methodology introduces additional uncertainty for some of the models during April and May (CNRM, MIROC-med, GFDL21).
- In light of this, the use of downscaled data from the significantly different GCMs to project future changes in the occurrence of *Salmonella* infections in April and May may lead to erroneous conclusions.

Following the chain of information from global climate models to the BCCA downscaled data (Figure 1), we investigated the characteristics of the GCM data re-gridded to 2° × 2° and subsequently bias-corrected (using a quantile mapping procedure). This illustrated that:

- The bias-correction corrects the large biases of the re-gridded GCM data and renders the temperature simulations viable for the evaluation of potential for *Salmonella* occurrences.
- The direct application of 2-degree re-gridded climate-model based indices is not advisable. The bias correction and downscaling obscure the knowledge on uncertainty in the GCM simulations.
- The characteristics of the variability of the statistically downscaled data and indices are inherited from the daily weather variability of the GCMs. The downscaling methods discussed here are not developed to correct for this variability. The sequences of weather patterns in global climate models are closely related to their abilities to represent various physical processes, such as blocking, that may bring about extended periods with high temperatures. Therefore, we draw attention to climate-model processes that need to be well represented to improve the salience of their application by practitioners.

In addition, to using daily statistically downscaled data directly for projecting potential future changes in *Salmonella* occurrences, it may be advisable for epidemiological practitioners to seek to develop relationships between *Salmonella* occurrences and period means and regional averages of HD30, which are also represented reasonably well by the downscaled GCMs. Though *Salmonella* epidemiological applications show sensitivity to sub-weekly to weekly weather events, the information from the 2-degree re-gridded climate models on local scales was inadequate to provide this level of detail. The bias-correction and downscaling employed here correct to a great extent these deficiencies and increase our confidence in using these data to project how the *Salmonella* incidences may change in the future in the height of the summer season. We have to be careful, however, when considering the potential for infections during periods of transition towards and after the warmest part of the year (namely during April, May and possibly September) using some of the downscaled GCMs.

In conclusion, evaluation of both GCM and downscaled GCM data is important to inform interpretation of future disease risk projections. Such evaluation should be tailored to the specific needs of an application, taking into account the scientific question and the geographic location. The credibility of the downscaled data depends both on the GCMs that serve as an input and on the statistical downscaling method, in our case. Assessment and provision of information to practitioners about the abilities of the GCMs to represent the meteorological processes that bring about the impacts affecting their areas of responsibility, in this case, public health, is critical and necessary step that needs to be considered. We recommend that a step-by-step analysis of the transformation of climate data throughout the chain of information from GCMs to downscaled outputs, similar to the one presented here, be used regularly to inform practitioners who assess vulnerability and risk of climate impacts and develop climate adaptation measures.

## Figures and Tables

**Table 1:** Temperature indices for evaluation of downscaled data in relation to *Salmonella* incidences; temporal scale for the index (week, month, year) depends on the application.

Index	Description
HD30	Number of “hot” days with daily maximum temperature (tasmax) >30 °C
HD35	Number of “hot” days with tasmax >35 °C
TR (tropical nights)	Number of “tropical” nights with daily minimum temperature (tasmin) >20 °C

**Table 2:** List of data used to calculate the heat indices.

Data	Abbreviation	Resolution
ARRM downscaled GCMs tasmax, tasmin 20C3M experiment	ARRM_ensemble_1/8	1/8° lat × 1/8° lon (approx.12 km)
BCCA downscaled GCMs tasmax, tasmin 20C3M experiment	BCCA_ensemble_1/8	same
Observed—Maurer02v2 tasmax, tasmin	Maurer02v2_1/8	same
Re-gridded GCM tasmax 20C3M experiment	GCM_2deg	2° lat × 2° lon
Bias-Corrected Re-gridded GCM tasmax 20C3M experiment	BC GCM_2deg	same
Re-gridded Observed—Maurer02v1 tasmax	Maurer02v1_2deg	same

**Table 3:** Comparisons performed between model derived data and observed data

Dataset	Maurer02v2_1/8	Maurer02v1_2deg
ARRM_ensemble_1/8	X	
BCCA_ensemble_1/8	X	
GCM_2deg		X
BC GCM_2deg		X

**Table 4:** Details about the GCMs used in the analyses.

CMIP3 Model i.d.	Country	Atmosphere Model Component—Horizontal Resolution lat × lon
CGCM3.1(T47)	Canada	3.75° × 3.75°
CNRM-CM3	France	2.8° × 2.8°
ECHAM5/MPI-OM	Germany	1.9° × 1.9°
ECHO-G	Germany/Korea	3.75° × 3.75°
GFDL-CM2.0	USA	2.0° × 2.5°
GFDL-CM2.1	USA	2.0° × 2.5°
MIROC3.2(medres)	Japan	approx. 2.8° × 2.8°
MRI-CGCM2.3.2	Japan	approx. 2.8° × 2.8°

## Supplementary Materials

The following are available online at www.mdpi.com/1660-4601/13/3/267/s1, Figure S1: Histograms of HD30 in July, 1971–2000, Washington DC area, as represented by the Maurer02v2_1/8 observed data and the individual downscaled GCM time series for the ARRM_ensemble_1/8, Figure S2: Histograms of HD30 in June, 1971–2000, Washington DC area, as represented by the Maurer02v2_1/8 observed data and the individual downscaled GCM time series for (a) the ARRM_ensemble_1/8 and (b) the BCCA_ensemble_1/8, Figure S3: Histograms of HD30 in August, 1971–2000, Washington DC area, as represented by the Maurer02v2_1/8 observed data and the individual downscaled GCM time series for (a) the ARRM_ensemble_1/8 and (b) the BCCA_ensemble_1/8, Figure S4: Histogram of HD30 in September, 1971–2000, Washington DC area, as represented by the Maurer02v2_1/8 observed data and the individual downscaled GCM time series for (a) the ARRMensemble_1/8 and (b) the BCCA_ensemble_1/8, Figure S5: Histograms of HD30 in April, 1971–2000, Washington DC area, as represented by the Maurer02v2_1/8 observed data and the individual downscaled GCM time series for (a) the ARRM_ensemble_1/8 and (b) the BCCA_ensemble_1/8, Figure S6: Histograms of HD30 in May, 1971–2000, Washington DC area, as represented by the Maurer02v2_1/8 observed data and the individual downscaled GCM time series for (a) the ARRM_ensemble_1/8 and (b) the BCCA_ensemble_1/8, Figure S7: Histograms of HD30 in (a) April and (b) May for a grid cell that overlays the Washington DC area, based on the individual 8 CMIP3 GCMs re-gridded to 2° × 2° resolution – GCM_2deg data compared to Maurer02v1_2deg, 1971–2000, Figure S8: Histograms of HD30 in (a) June and (b) August for a grid cell that overlays the Washington DC area, based on the individual 8 CMIP3 GCMs re-gridded to 2° × 2° resolution – GCM_2deg data compared to Maurer02v1_2deg, 1971–2000, Figure S9: Histogram of HD30 in September for a grid cell that overlays the Washington DC area, based on the individual 8 CMIP3 GCMs re-gridded to 2° × 2° resolution—GCM_2deg data compared to Maurer02v1_2deg, 1971–2000, Table S1: Medians of index values, absolute biases (downscaled—observed data), and percent biases ((absolute bias/observed data) ×100), based on the ARRM and BCCA downscaled ensembles (the results are reported as ARRM value / BCCA value), for all areas and all months April–September for 1971–2000. The index values for the Maurer02v2_1/8 observed data used as the baseline for the comparisons are included as well, Table S2: Statistical significance (*p*-values) from the Brunner-Munzel (B–M) test for stochastic equality applied to the monthly HD 30 distributions of downscaled GCM data compared to the Maurer02v2 data, as well as applied to the Bias-Corrected GCM, and the Re-Gridded GCM data compared to the Maurer02v1 2° re-gridded data. Statistically significant results are indicated by bold font.
