# Supplementary material for: Evaluating the Appropriateness of Downscaled Climate Information for Projecting Risks of Salmonella
Source: Int J Environ Res Public Health. 2016 Feb 29;13(3):267. doi: 10.3390/ijerph13030267 (PMC4808930; doi:10.3390/ijerph13030267)
Supplement: Supplementary file 1 [file ijerph-13-00267-s001.pdf]

Supplementary Materials: Evaluating the Appropriateness of Downscaled Climate Information for Projecting Risks of Salmonella

Galina S. Guentchev, Richard B. Rood, Caspar M. Ammann, Joseph J. Barsugli, Kristie Ebi, Veronica Berrocal, Marie S. O'Neill, Carina J. Gronlund, Jonathan L. Vigh, Ben Koziol and Luca Cinquini

Table S1. Medians of index values, absolute biases (downscaled—observed data), and percent biases ((absolute bias/observed data) × 100), based on the ARRM and BCCA downscaled ensembles (the results are reported as ARRM value/BCCA value), for all areas and all months April–September for 1971–2000. The index values for the Maurer02v2_1/8 observed data used as the baseline for the comparisons are included as well.

Area, Index	Month	Maurer02v2_1/8 Value	Median Value	Median Absolute Bias	Median Percent Bias
1—Washington DC area, HD30 Mean	April	0.8	0.4/0.3	−0.4/−0.5	−50.0/−62.5
	May	3.1	2.4/2.1	−0.7/−1.0	−22.6/−32.2
	June	10.5	10.8/10.5	0.3/0	2.9/0
	July	19.0	19.2/20.2	0.2/1.2	1.1/6.3
	August	15.2	17.2/15.7	2.0/0.5	13.2/3.3
	September	5.2	5.5/5.1	0.3/-0.1	5.8/−1.9
2010—SE MI Climate Division, HD30 Mean	April	0.1	0/0	−0.1/−0.1	−100.0/−100.0
	May	1.5	0.8/0.7	−0.7/−0.8	−46.7/−53.3
	June	5.1	4.8/4.6	−0.3/−0.5	−5.6/−9.8
	July	9.1	9.1/9.3	0/0.2	0/2.2
	August	5.3	7.0/5.1	1.7/−0.2	22.6/−3.8
	September	1.7	1.6/1.4	−0.1/−0.3	−5.6/−17.6
3110—Wayne County MI, HD30 Mean	April	0.1	0.1/0	0/−0.1	0/−100.0
	May	1.8	0.9/0.9	−0.9/−0.9	−50.0/−50.0
	June	5.7	5.7/5.6	0/−0.1	0/−1.8
	July	10.6	10.4/11.1	−0.2/0.5	−1.9/4.7
	August	6.6	8.4/6.6	1.8/0	27.3/0
	September	2.1	2.1/1.7	0/−0.4	0/−19.0
3175—Cuyahoga County OH, HD30 Mean	April	0.1	0/0	−0.1/−0.1	−100.0/−100.0
	May	1.0	0.6/0.6	−0.4/−0.4	−40.0/−40.0
	June	4.3	4.1/3.9	−0.2/−0.4	−4.7/−9.3
	July	8.1	7.9/8.2	−0.2/0.1	−2.5/1.2
	August	4.9	6.4/4.4	1.5/−0.5	30.6/−10.2
	September	1.6	1.5/1.5	−0.1/−0.1	−6.3/−6.3

Table S1. *Cont.*

Area, Index	Month	Maurer02v2_1/8 Value	Median Value	Median Absolute Bias	Median Percent Bias
3303—NE OH Climate Division, HD30 Mean	April	0.1	0/0	−0.1/−0.1	−100.0/−100.0
	May	0.9	0.5/0.6	−0.4/−0.3	−44.4/−33.3
	June	3.9	3.9/3.6	0/−0.3	0/−7.7
	July	7.6	7.6/7.7	0/0.1	0/1.3
	August	4.6	6.2/4.2	1.6/−0.4	34.8/−8.7
	September	1.4	1.6/1.3	0.2/−0.1	14.3/−7.1
1—Washington DC area, HD35 Mean	April	0	0/0	0/0	0/0
	May	0	0/0	0/0	0/0
	June	0.5	0.6/0.2	0.1/−0.3	20.0/−60.0
	July	2.6	2.1/1.2	−0.5/−1.4	−19.2/−53.8
	August	1.3	1.5/0.4	0.2/−0.9	15.4/−69.2
	September	0.3	0.2/0.1	−0.1/−0.2	−33.3/−66.7
2010—SE MI Climate Division, HD35 Mean	April	0	0/0	0/0	0/0
	May	0	0/0	0/0	0/0
	June	0.3	0.1/0.1	−0.2/−0.2	−66.7/−66.7
	July	0.6	0.4/0.2	−0.2/−0.4	−33.3/−66.7
	August	0.2	0.2/0	0/−0.2	0/−100.0
	September	0	0/0	0/0	0/0
3110—Wayne County MI, HD35 Mean	April	0	0/0	0/0	0/0
	May	0	0/0	0/0	0/0
	June	0.3	0.2/0.1	−0.1/−0.2	−33.3/−66.7
	July	0.6	0.5/0.3	−0.1/−0.3	−16.7/−50.0
	August	0.2	0.3/0.1	0.1/−0.1	50.0/−50.0
	September	0	0/0	0/0	0/0
3175—Cuyahoga County OH, HD35 Mean	April	0	0/0	0/0	0/0
	May	0	0/0	0/0	0/0
	June	0.1	0.1/0	0/−0.1	0/−100.0
	July	0.4	0.2/0.1	−0.2/−0.3	−50.0/−75.0
	August	0.1	0.1/0	0/−0.1	0/−100.0
	September	0	0/0	0/0	0/0

Table S1. *Cont.*

Area, Index	Month	Maurer02v2_1/8 Value	Median Value	Median Absolute Bias	Median Percent Bias
3303—NE OH Climate Division, HD35 Mean	April	0	0/0	0/0	0/0
	May	0	0/0	0/0	0/0
	June	0.1	0.1/0	0/−0.1	0/−100.0
	July	0.3	0.1/0.1	−0.2/−0.2	−66.7/−66.7
	August	0.1	0.1/0	0/−0.1	0/−100.0
	September	0	0/0	0/0	0/0
1—Washington DC area, TR Mean	April	0	0/0	0/0	0/0
	May	0.3	0.2/0.1	−0.1/−0.2	−33.3/−66.7
	June	4.6	3.8/2.6	−0.8/−2.0	−17.4/−43.5
	July	12.2	11.7/11.4	−0.5/−0.8	−4.1/−6.6
	August	9.2	9.8/7.8	0.6/−1.4	6.5/−15.2
	September	2.2	2.4/1.3	0.2/−0.9	9.1/−40.9
2010—SE MI Climate Division, TR Mean	April	0	0/0	0/0	0/0
	May	0.2	0.1/0	−0.1/−0.2	−50.0/−100.0
	June	1.4	1.5/1.2	0.1/−0.2	7.1/−14.3
	July	4.5	4.1/4.0	−0.4/−0.5	-8.9/−11.1
	August	2.5	3.0/2.2	−0.5/−0.3	20.0/−12.0
	September	0.5	0.5/0.4	0/−0.1	0/−20.0
3110—Wayne County MI, TR Mean	April	0	0/0	0/0	0/0
	May	0.3	0.1/0.1	−0.2/−0.2	−66.7/−66.7
	June	2.1	2.2/1.8	0.1/−0.3	4.8/−14.3
	July	6.5	5.9/5.9	−0.6/−0.6	−9.2/−9.2
	August	4.2	4.6/3.5	0.4/−0.7	9.5/−16.7
	September	0.8	0.9/0.7	0.1/−0.1	12.5/−12.5
3175—Cuyahoga County OH, TR Mean	April	0	0/0	0/0	0/0
	May	0.2	0.1/0	−0.1/−0.2	−50.0/−100.0
	June	1.7	1.7/1.3	0/−0.4	0/−23.5
	July	5.6	4.9/4.4	−0.7/−1.2	−12.5/−21.4
	August	3.0	3.5/2.5	0.5/−0.5	16.7/−16.7
	September	0.9	0.7/0.4	−0.2/−0.5	−22.2/−55.6

Table S1. *Cont.*

Area, Index	Month	Maurer02v2_1/8 Value	Median Value	Median Absolute Bias	Median Percent Bias
3303—NE OH Climate Division, TR Mean	April	0	0/0	0/0	0/0
	May	0.2	0/0	−0.2/−0.2	−100.0/−100.0
	June	1.1	1.1/0.8	0/−0.3	0/−27.3
	July	3.7	3.4/2.8	−0.3/−0.9	−8.1/−24.3
	August	2.0	2.2/1.5	0.2/−0.5	10.0/−25.0
	September	0.5	0.4/0.3	−0.1/−0.2	−20.0/−40.0

Table S2. Statistical significance (*p*-values) from the Brunner-Munzel (B—M) test for stochastic equality applied to the monthly HD 30 distributions of downscaled GCM data compared to the Maurer02v2 data, as well as applied to the Bias-Corrected GCM, and the Re-Gridded GCM data compared to the Maurer02v1 2° re-gridded data. Statistically significant results are indicated by bold font.

Downscaling Method	GCM	April	May	June	July	August	September
ARRM	CGCM3	0.559	0.598	0.785	0.721	0.692	0.087
	CNRM	**0.005**	**0.015**	0.92	0.105	0.262	**0.013**
	ECHAM5	0.493	0.891	0.382	0.942	0.179	0.862
	ECHOG	**0.021**	0.091	0.111	0.885	0.136	0.887
	GFDL20	0.126	0.559	0.966	0.896	0.31	0.458
	GFDL21	0.258	**0.031**	0.874	0.423	0.831	0.977
	MIROCMed	**0.01**	0.144	0.245	0.819	0.176	0.622
	MRICGCM2	**0.002**	0.570	0.908	0.414	0.245	0.258
BCCA	CGCM3	0.166	0.098	0.717	0.339	0.58	0.555
	CNRM	**0.004**	0.282	0.966	0.178	0.416	0.695
	ECHAM5	0.258	0.312	0.966	0.794	0.886	0.652
	ECHOG	0.352	0.459	0.333	0.465	0.796	0.749
	GFDL20	0.173	0.342	0.831	0.508	0.68	0.465
	GFDL21	**0.044**	**0.026**	0.92	0.379	0.795	0.965
	MIROCMed	0.093	**0.023**	0.616	0.438	0.645	0.885
	MRICGCM2	0.143	0.071	0.409	0.493	0.581	0.828

Table S2. *Cont.*

GCM Data	GCM	April	May	June	July	August	September
GCM 2 deg Bias Corrected Data	CGCM3	0.864	0.424	0.546	0.93	0.924	0.152
	CNRM	0.133	0.681	0.676	0.445	0.238	0.781
	ECHAM5	0.833	0.458	0.503	0.823	0.526	0.69
	ECHOG	0.672	0.552	0.523	0.715	0.607	0.934
	GFDL20	0.34	0.527	0.948	0.864	0.678	0.13
	GFDL21	0.098	0.618	0.836	0.677	0.318	0.988
	MIROCMed	0.469	0.884	0.396	0.911	0.802	0.701
	MRICGCM2	0.818	0.639	0.947	0.431	0.654	0.112
GCM 2 deg re-gridded	CGCM3	**0.02**	0.193	**0.02**	**0.0004**	0.076	0.378
	CNRM	**0.006**	**2.374e-07**	**<2.2e-16**	NA	NA	**3.347e-12**
	ECHAM5	**0.006**	**0.000003**	**0.0006**	**4.349e-10**	0.087	**0.015**
	ECHOG	**0.006**	**2.374e-07**	**<2.2e-16**	NA	NA	**3.347e-12**
	GFDL20	**0.006**	**2.374e-07**	**<2.2e-16**	NA	NA	**3.347e-12**
	GFDL21	**0.006**	**2.374e-07**	**<2.2e-17**	**4.44e-16**	**2.054e-08**	**0.000002**
	MIROCMed	**0.006**	**0.0003**	**0.013**	**<2.2e-16**	**<2.2e-16**	**1.924e-10**
	MRICGCM2	**0.006**	**0.003**	**6.725e-05**	**4.649e-06**	**0.000008**	**0.008**

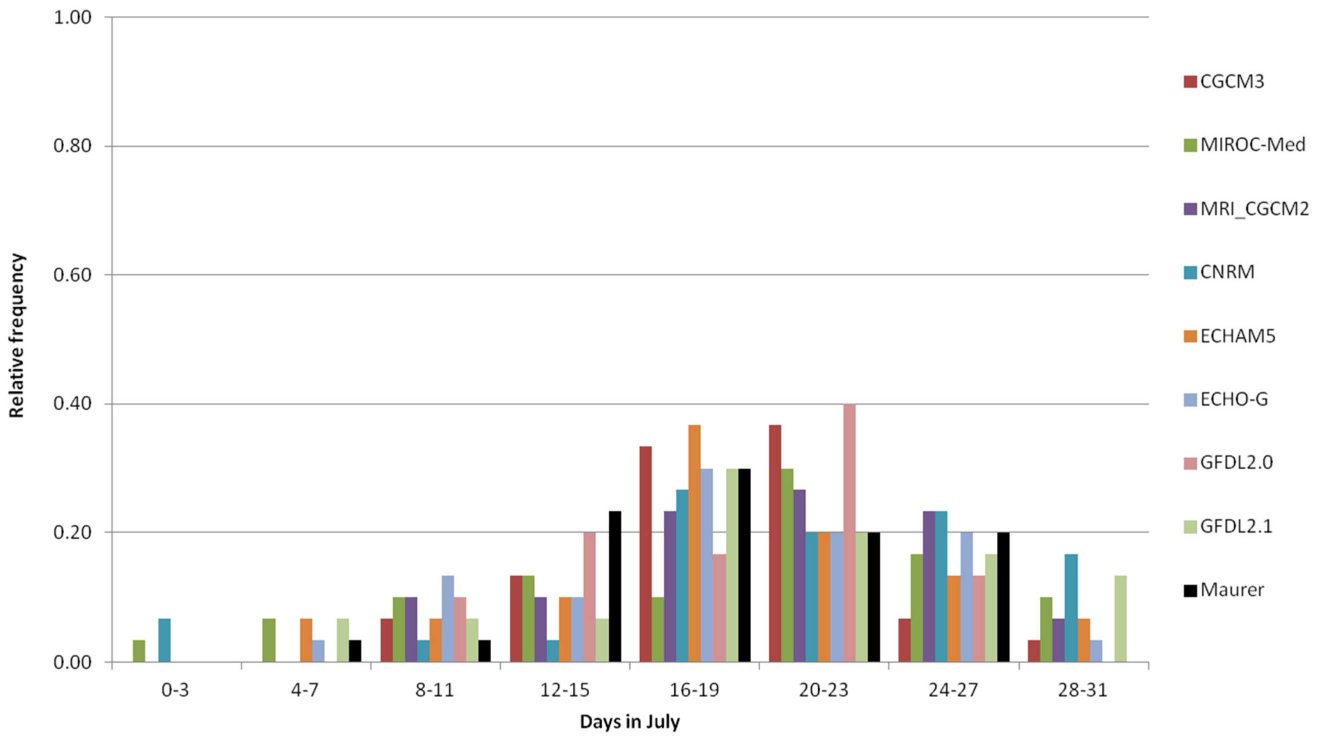

Figure S1. Histogram of HD30 in July, 1971–2000, Washington DC area, as represented by the Maurer02v2_1/8 observed data and the individual downscaled GCM time series for the ARRM_ensemble_1/8.

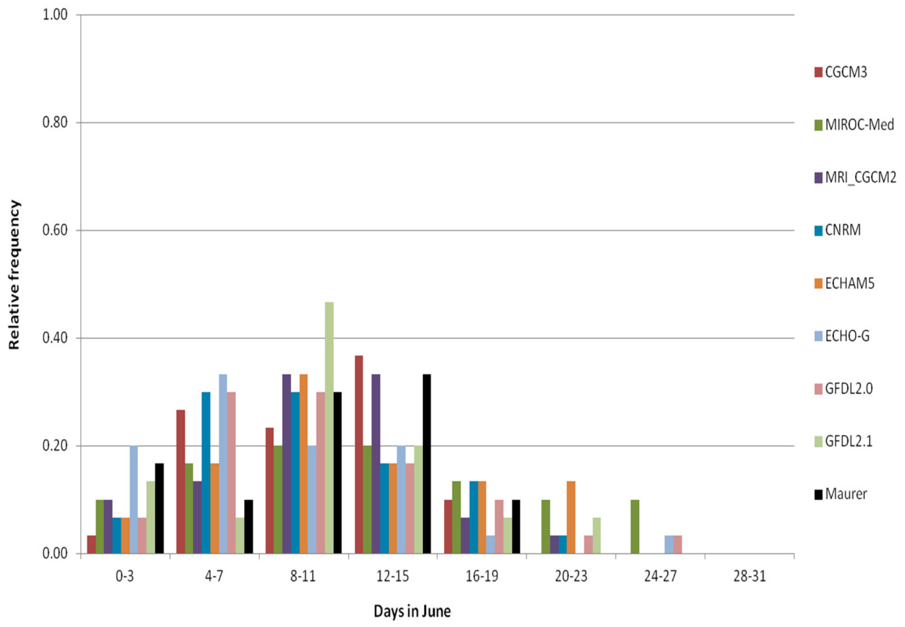

(**a**)

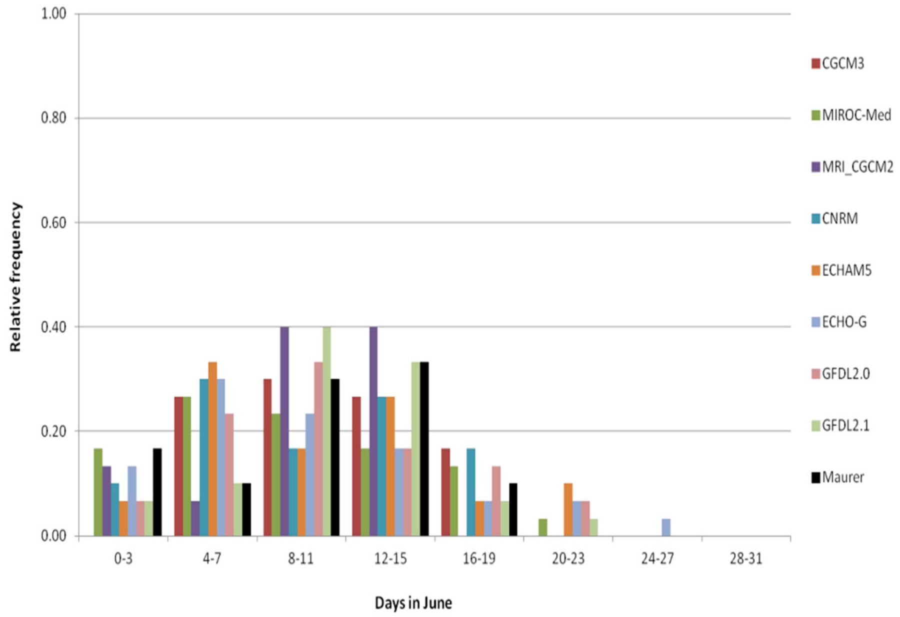

(**b**)

Figure S2. Histograms of HD30 in June, 1971–2000, Washington DC area, as represented by the Maurer02v2_1/8 observed data and the individual downscaled GCM time series for (**a**) the ARRM_ensemble_1/8 and (**b**) the BCCA_ensemble_1/8.

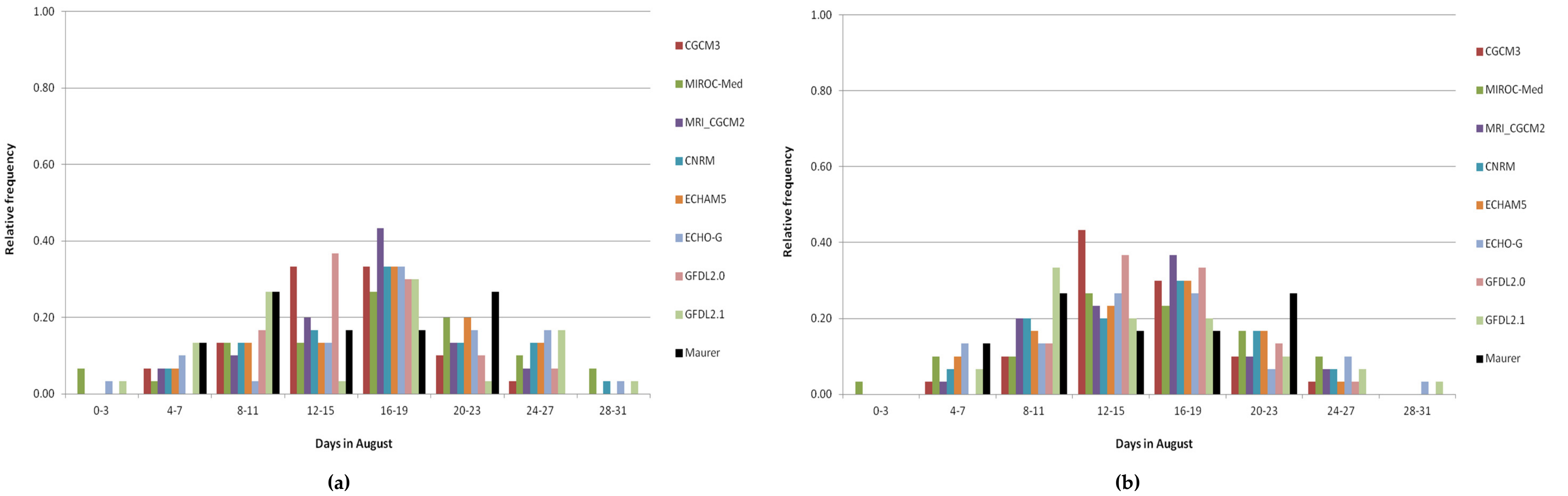

Figure S3. Histograms of HD30 in August, 1971–2000, Washington DC area, as represented by the Maurer02v2_1/8 observed data and the individual downscaled GCM time series for (**a**) the ARRM_ensemble_1/8 and (**b**) the BCCA_ensemble_1/8.

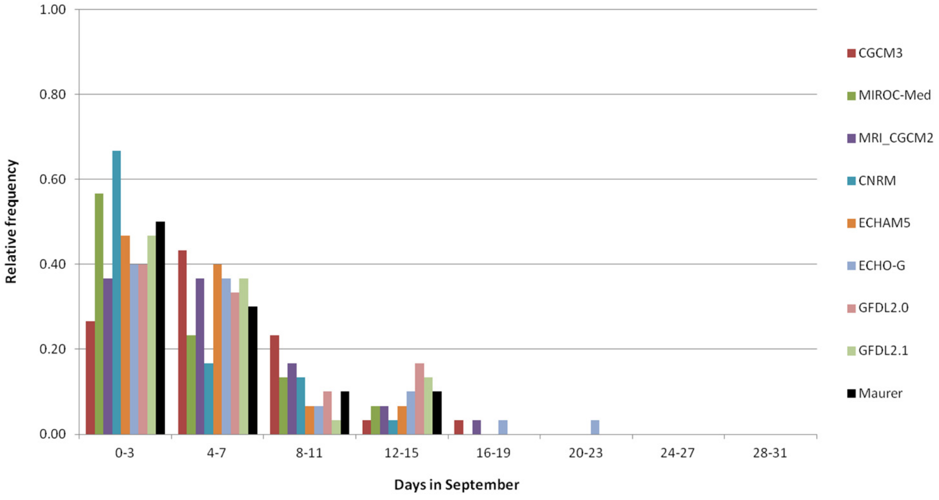

(**a**)

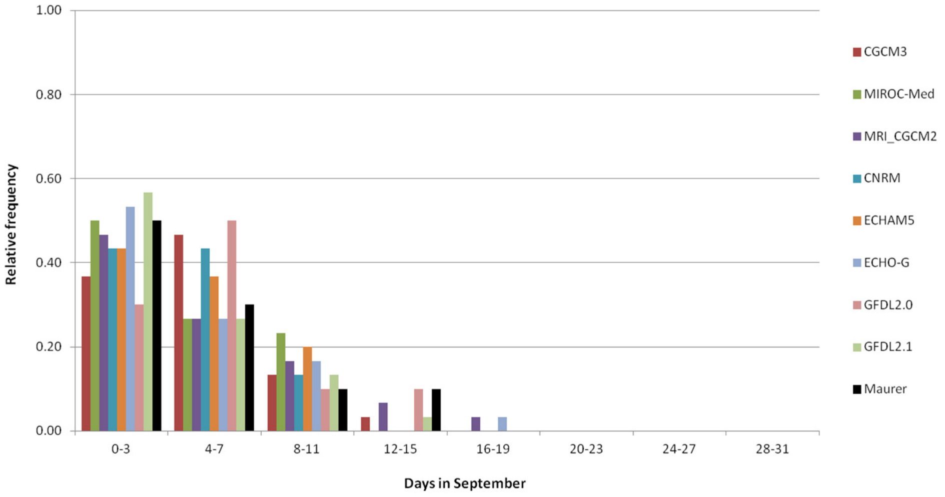

(**b**)

Figure S4. Histograms of HD30 in September, 1971–2000, Washington DC area, as represented by the Maurer02v2_1/8 observed data and the individual downscaled GCM time series for (**a**) the ARRM_ensemble_1/8 and (**b**) the BCCA_ensemble_1/8.

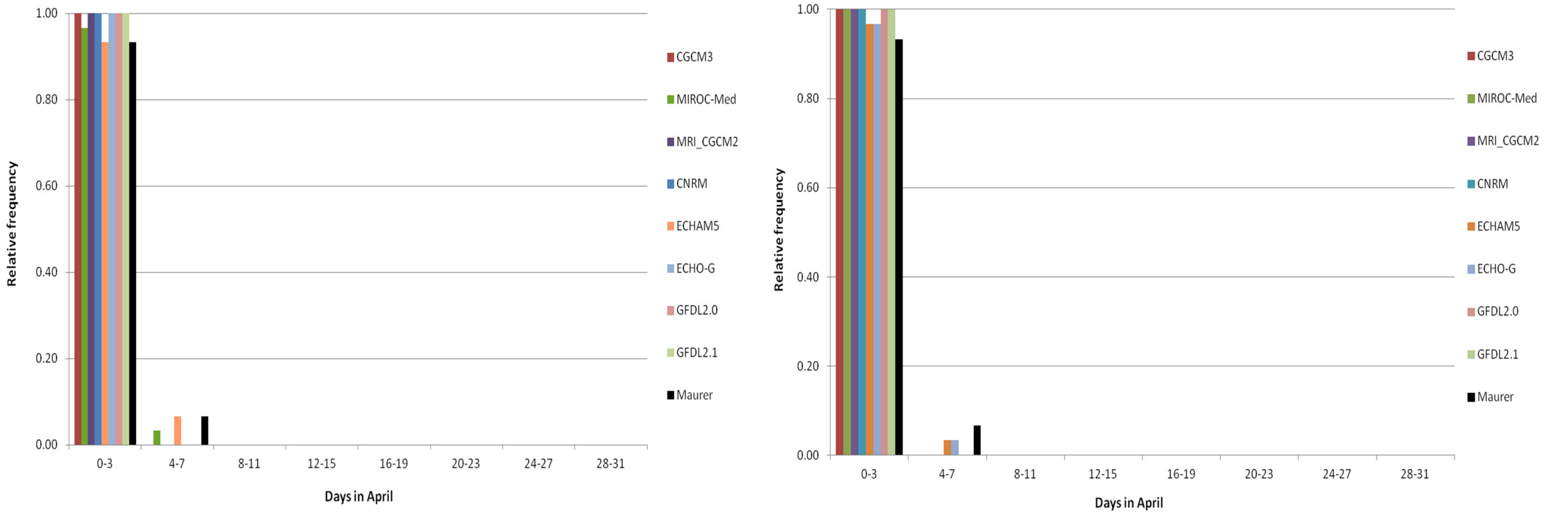

(**a**) (**b**)

Figure S5. Histograms of HD30 in April, 1971–2000, Washington DC area, as represented by the Maurer02v2_1/8 observed data and the individual downscaled GCM time series for (**a**) the ARRM_ensemble_1/8 and (**b**) the BCCA_ensemble_1/8.

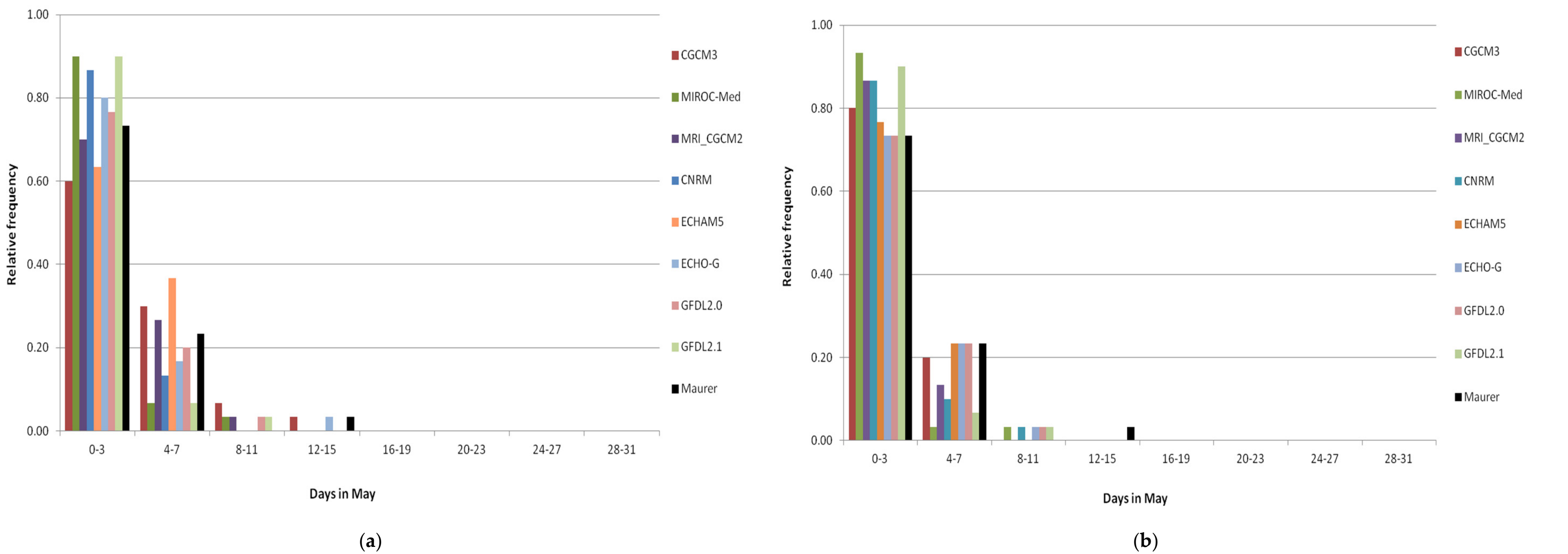

Figure S6. Histograms of HD30 in May, 1971–2000, Washington DC area, as represented by the Maurer02v2_1/8 observed data and the individual downscaled GCM time series for (**a**) the ARRM_ensemble_1/8 and (**b**) the BCCA_ensemble_1/8.

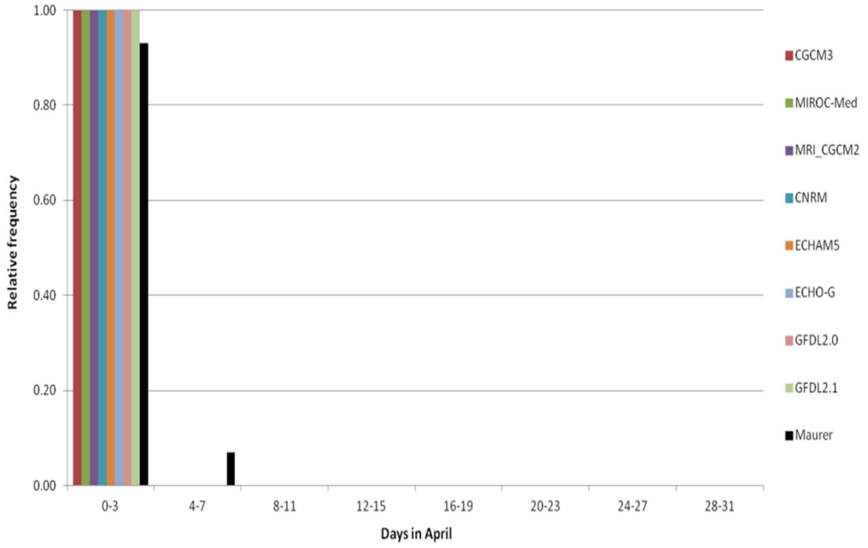

(**a**)

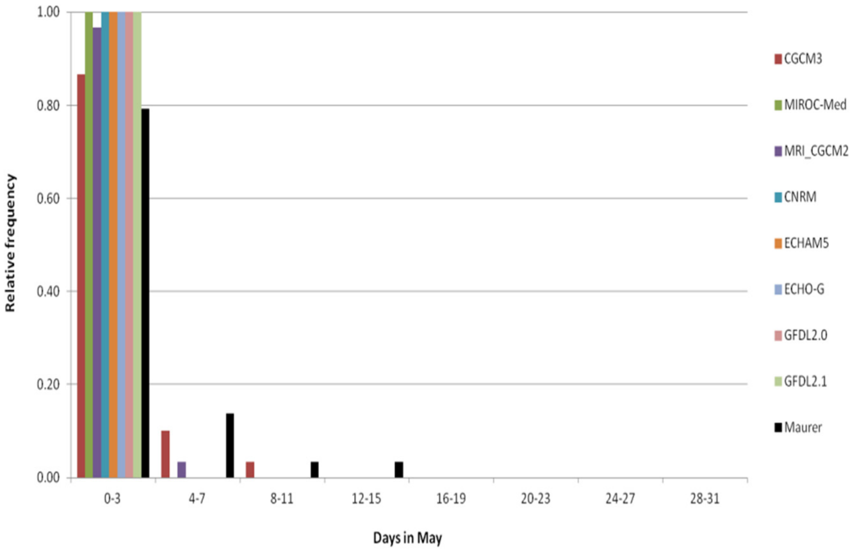

(**b**)

Figure S7. Histograms of HD30 in (**a**) April and (**b**) May for a grid cell that overlays the Washington DC area, based on the individual 8 CMIP3 GCMs re-gridded to 2° × 2° resolution—GCM_2deg data compared to Maurer02v1_2deg, 1971–2000.

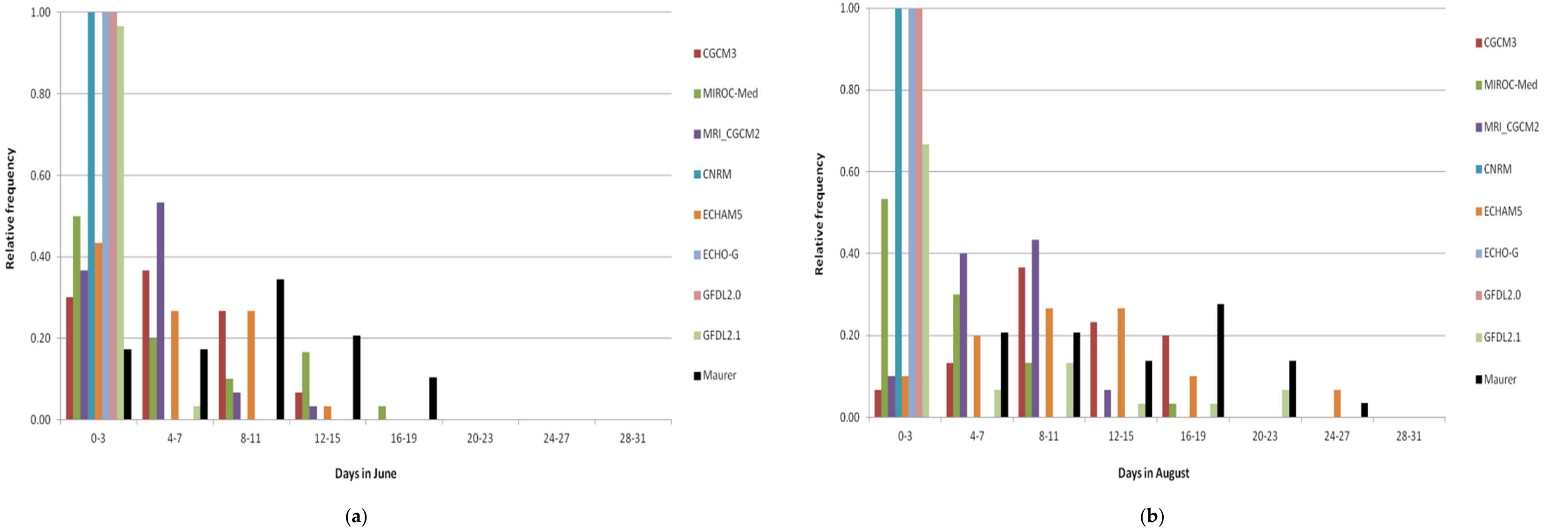

Figure S8. Histograms of HD30 in (**a**) June and (**b**) August for a grid cell that overlays the Washington DC area, based on the individual 8 CMIP3 GCMs re-gridded to 2° × 2° resolution—GCM_2deg data compared to Maurer02v1_2deg, 1971–2000.

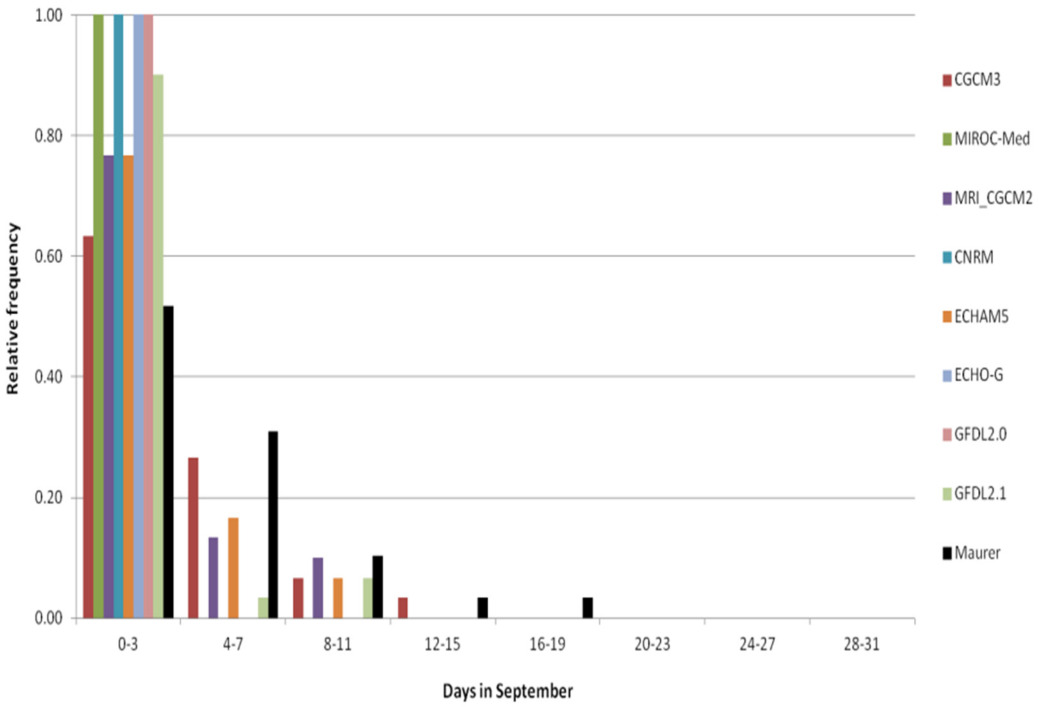

Figure S9. Histogram of HD30 in September for a grid cell that overlays the Washington DC area, based on the individual 8 CMIP3 GCMs re-gridded to 2° × 2° resolution—GCM_2deg data compared to Maurer02v1_2deg, 1971–2000.

© 2016 by the authors; licensee MDPI, Basel, Switzerland. This article is an open access article distributed under the terms and conditions of the Creative Commons by Attribution (CC-BY) license (http://creativecommons.org/licenses/by/4.0/).
